# Quality of Life in Long-Standing Rheumatoid Arthritis: What Can Help Apart from Treatment? A Single-Center Cross-Sectional Observational Study

**DOI:** 10.3390/jcm14248925

**Published:** 2025-12-17

**Authors:** Bogna Grygiel-Górniak, Ewelina Kowynia, Mohamed Abouzid, Anna Kwaśniewska, Maria Joks, Natalia Majewska, Włodzimierz Samborski

**Affiliations:** 1 Department of Rheumatology, Rehabilitation and Internal Diseases, Poznan University of Medical Sciences, 61-545 Poznań, Poland; 2 Department of Physical Pharmacy and Pharmacokinetics, Faculty of Pharmacy, Poznan University of Medical Sciences, 60-806 Poznań, Poland; 3 Rheumatological Centre in Śrem, 63-100 Śrem, Poland; 4 Rheumatology Research Group, Department of Rheumatology, Rehabilitation and Internal Diseases, Poznan University of Medical Sciences, 61-545 Poznań, Poland; 5 Department of Clinical Radiology, Poznan University of Medical Sciences, 61-848 Poznań, Poland

**Keywords:** rheumatoid arthritis, quality of life, WHO-BREF, depression, social relationships

## Abstract

**Background:** Rheumatoid arthritis (RA) is characterized by pain, limited mobility, an increased risk of depression, and sleep disturbances, which significantly impact quality of life (QoL). Analyzing psychosocial and environmental factors can identify the most significant determinants of QoL in RA patients. **Methods:** We conducted a single-center cross-sectional observational study among RA patients with low disease activity (DAS28ESR ≤ 3.2) from September 2021 till May 2022 in a rheumatology clinic or biology ward during one-day follow-up visits. QoL in RA patients was analyzed using the World Health Organization Quality of Life Instrument, Short Form (WHOQOL-BREF), the Beck Depression Inventory (BDI), and the Pittsburgh Sleep Quality Index (PSQI) using a backward stepwise multivariate regression model (heatmap). **Results:** A total of 116 patients were included, predominantly overweight females (76.7%), with a median disease duration of 19 years. Only 24% of patients had good sleep quality, while approximately 20% had risk factors for moderate to severe depression. WHOQOL-BREF scores indicated moderate physical health (median = 50), high psychological well-being (66.7), strong social relationships (75), a supportive environment (68.8), good overall QoL (75), and average overall health (50). The multivariate model revealed that younger patients (under 40 years of age), married individuals, and those with good sleep quality (as indicated by a low PSQI) had the highest scores in the various WHO-BREF domains. The physical activity level was positively correlated with overall QoL (*p* < 0.001) and overall health (*p* = 0.017). BMI and BDI values were negatively associated (pBMI < 0.05; pBDI < 0.001) with physical health (rBMI = −0.34; rBDI = −0.73), social relationships (rBMI = −0.19; rBDI = −0.73), psychological well-being (rBMI = −0.24; rBDI = −0.83), and overall health (rBMI = −0.26; rBDI = −0.46). **Conclusions:** This study shows that, in addition to appropriate treatment, maintaining good physical health, satisfying relationships, and ensuring quality sleep are crucial for improving the QoL of patients with RA.

## 1. Introduction

RA is an inflammatory joint disease characterized by chronic, progressive inflammation of the synovial membrane, leading to joint damage and involvement of periarticular elements. The overall worldwide prevalence of RA ranges from 0.24 to 1%, although rates vary by country and geographic region [1]. RA develops mainly in young people (between 30 and 50 years of age), but due to its chronic course, a large group of patients are elderly patients. Despite treatment, the disease is chronic with recurrent exacerbations, often leading to irreversible joint deformation. The progressive nature of RA is the leading cause of disability in about 30% of patients, which undoubtedly affects their QoL [2,3].

Most patients with RA experience fatigue, and about 15% report severe fatigue, which is accompanied by chronic pain and decreased mood, affecting daily functioning [4]. Fatigue co-occurs and correlates with pain, poor sleep, obesity, and comorbidities [5,6]. Moreover, there is a direct correlation between the QoL (as measured by subjective feelings of health and functional capacity) and RA activity [7]. Hence, proper treatment of RA includes not only appropriate pharmacotherapy but also exercise programs and behavioral interventions (including supervised self-management programs with cognitive behavioral therapy, mindfulness, and reinforcement such as reminders) [8].

Considering the above data, we intend to assess which of the analyzed socio-demographic factors, as well as the symptoms of the disease (pain, fatigue, progressive disability) characteristic of RA, affect the QoL and may influence the treatment outcomes. Based on the collected data and study design, we sought to identify factors that most significantly impact health perception in hospitalized patients with long-term rheumatoid arthritis. High disease activity can significantly impact patients’ QoL. To eliminate this factor, we analyzed patients with low disease activity or in remission who were hospitalized in a rheumatology clinic or biology ward during one-day follow-up visits. We intended to determine how patients assess their QoL depending on various variables (including age, duration of the disease, physical activity, social relationships, etc.). The current research should identify the healthcare and educational aspects that may be considered when planning long-term treatment for patients with this disease.

## 2. Materials and Methods

### 2.1. Study Design and Setting

The data for this study were obtained from a single-center, cross-sectional, observational, non-interventional study involving consultations with patients with RA hospitalized in a rheumatology department (a rheumatology clinic or biology ward) during one-day follow-up visits between September 2021 and May 2022. In this study, patients completed self-administered questionnaires, with measures taken to ensure the accuracy and reliability of responses. All participants were informed that their responses would remain confidential. A trained facilitator was present to provide guidance and clarify any questions regarding the completion of the questionnaires.

### 2.2. Sample

Patients were invited to participate in the study (n = 176) during their standard hospitalization at the rheumatology clinic or biologic department (one-day control visit). The diagnosis of RA was made according to the 2010 American College of Rheumatology/European League Against Rheumatism criteria. We conducted an in-person survey, and patient disease activity (clinical information) was assessed by rheumatologists rather than the patients themselves, thereby avoiding inclusion and recall bias. Since high disease activity may influence QoL, in all domains (including physical, psychological, environment, and social domains) [9,10], we decided to select patients with low disease activity or remission, which were assessed using the Disease Activity Score 28-Erythrocyte Sedimentation Rate (DAS28-ESR) with a value of ≤3.2 defined as the threshold for a low disease activity state and <2.6 as the threshold for remission [11]. Therefore, we aimed to assess the factors, unrelated to high disease activity, that influence quality of life. Such an analysis should enable us to identify which factors may influence QoL in patients with a low DAS28. In our cohort, patients with a DAS28 score greater than 3.2. (n = 39), or patients who did not complete all the study documentation (n = 21) were excluded from the analysis. Finally, 116 patients were included in the analysis.

#### 2.2.1. Sample Size Considerations

Because this was an exploratory cross-sectional study, the sample size was determined pragmatically by including all eligible patients during the study period. Although no formal a priori power calculation was performed, the final sample of 116 participants meets the commonly recommended methodological criteria for multivariable regression analyses (at least 10–15 participants per predictor variable), providing sufficient power to detect small to moderate associations. In addition, our sample size is consistent with other published QoL studies in rheumatoid arthritis, which typically include approximately 100–120 patients.

#### 2.2.2. Procedures

Clinical data were obtained from patients’ medical records during their hospital or biological therapy department visit. Demographic data (including age, sex, and ethnicity) and treatment information were available for all patients. All of them were treated with conventional synthetic DMARDs (csDMARDs) and/or biological DMARDs (bDMARDs). All patients were measured (body mass and height) by a nurse trained to carry out the tasks within the study. The role of the nurse was also to provide assistance to patients and offer explanations for any questions that were incomprehensible. The main objective was to obtain questionnaires that the patients had completed themselves. The study excluded individuals who had difficulty cooperating while completing the questionnaires.

All patients signed an informed consent form. The study was conducted in accordance with the ethical standards outlined in the 1975 Declaration of Helsinki and its subsequent amendments, or with comparable ethical standards. The study was approved by the relevant Bioethics Committee at the Poznań University of Medical Sciences (approval number KB-194/25). Written informed consent was obtained from all individual participants included in the study, and all patients provided written informed consent regarding their medical management and the use of medical data during their hospital visit.

#### 2.2.3. Inclusion and Exclusion Criteria

An observational, real-life study was conducted on 176 RA patients hospitalized in the rheumatology clinic. The study included all patients aged 18 years or older with RA, confirmed by a rheumatologist according to the classification criteria established by the American College of Rheumatology (ACR) and the European League Against Rheumatism (EULAR) [12]. Inclusion criteria were (1) meeting ACR/EULAR 2010 classification criteria for RA (including joint involvement, serology, acute-phase reactants, and symptom duration); patients who were RA diagnosed before 2010 had a confirmed diagnosis by clinical symptoms, serology analyses, and X-ray changes typical for RA (2) age ≥ 18; and (3) a minimum of 6 months follow-up in the rheumatology clinic or biologic therapy center; (4) treatment according to actual standards; (5) voluntary provision of informed consent to participate in the study; (6) low disease activity or remission (DAS28(ESR) ≤ 3.2) to exclude the possible influence of pain on QoL, sleep duration and depressive mood (7) ability to complete the study questionnaires independently.

Patients were excluded from the study when they were younger than 18 years or were diagnosed with chronic conditions that may interfere with the main results and QoL assessment, such as NYHA class IV heart failure, cancer, and severe liver or renal failure. Furthermore, subjects with mental disorders treated pharmacologically (depression, bipolar disorder, or other cognitive conditions involving low mood) or with no possibility to fill out the study questionnaires independently were also excluded from the study.

Patients underwent a review of medical records, a structured clinical interview, and a physical examination. Patients were assessed regarding their socioeconomic and demographic characteristics, smoking status, body mass index (BMI), RA treatment, complications, and disease duration. All patients were Caucasians. To assess disease activity, the chosen scores were the Disease Activity Score 28 with ESR (DAS28-ESR), and patients were treated according to the current EULAR/ACR recommendations [13].

### 2.3. Instruments

This study utilized the World Health Organization Quality of Life Instrument, Short Form (WHOQOL-BREF), a condensed version of the WHOQOL-100. The WHOQOL-BREF consists of 26 items: two assess the overall QoL and general health, while the remaining 24 measure satisfaction across four domains. These domains include Physical Health (7 items, DOM1), Psychological Health (6 items, DOM2), Social Relationships (3 items, DOM3), and Environmental Health (8 items, DOM4). Each item is rated on a 5-point Likert scale, with scores ranging from 1 (very poor/dissatisfied) to 5 (very good/satisfied).

The Polish version of the WHOQOL-BREF questionnaire was administered to 116 patients with rheumatoid arthritis. Raw scores for each domain were calculated by averaging the item scores within that domain. The resulting mean scores were then transformed into a 4–20 scale according to the WHOQOL-BREF scoring guidelines [14,15] (average domain scores × 4). Finally, these scores were linearly converted to a 0–100 scale; for example, domXb = (DOMX − 4) × (100/16). Only overall health and quality scores were converted using the formula f1b or f2b = (score − 1) × (100/4). More information is available in the WHOQOL-BREF Syntax files [14]. Higher scores indicate a better quality of life. The scales’ internal consistency reliability was determined with Cronbach’s standardized alpha [16].

The Beck Depression Inventory (BDI) is a 21-item, self-report rating inventory that measures characteristic attitudes and symptoms of depression [17]. Each item was scored 0 to 3 points, resulting in a total score range of 0 to 63. According to the OHSU Headache Center [18], Beck’s criteria for depression levels classify scores of 1 to 10 as normal, 11 to 16 as mild, 17 to 20 as borderline clinical depression, 21 to 30 as moderate depression, 31 to 40 as severe depression, and over 40 as extreme depression.

Sleep pattern and quality: participants were assessed on their sleep quality using the Pittsburgh Sleep Quality Index (PSQI) [19,20]. The PSQI was used to retrospectively assess sleep quality and disturbances over a 30-day period. The index comprised seven fundamental subdomains of sleep: (1) subjective sleep quality, (2) sleep latency, (3) sleep duration, (4) habitual sleep efficiency, (5) sleep disturbances, (6) use of sleep medications, and (7) daytime dysfunction, rated from 0 (indicating optimal sleep quality) to 3 (indicating poorer sleep quality). The sum of 7 components yields an overall score ranging from 0 to 21, with a cutoff value of 5. A score of less than 5 indicated good overall sleep quality.

### 2.4. Statistical Analysis

We performed the statistical analysis using PQStat Software (2021) v.1.8.2.238 (PQStat Software, Poznan, Poland). Listwise deletion was applied for missing data. The Shapiro–Wilk test was used to assess the normality of continuous data. Categorical data were reported as frequencies/percentages. In contrast, continuous data were presented as mean/standard deviation (SD) for normally distributed variables or median/interquartile range (IQR) for non-normally distributed variables. The Mann–Whitney U test was used to compare differences between two independent groups with non-normal distributions, whereas the *t*-test was applied when a normal distribution was present. The Kruskal–Wallis ANOVA was used to assess differences among independent groups with non-normal distributions, followed by Dunn-Bonferroni post hoc tests. For normally distributed data, one-way ANOVA was conducted, followed by Fisher’s LSD post hoc tests. Additionally, Spearman’s rank correlation coefficient (r) was used to examine correlations between body mass index, minutes spent outdoors, the number of daily meals, and the year rheumatoid arthritis started with the domains of environment, physical health, psychological well-being, social relationships, overall health, and overall QoL. Finally, we built a multivariate backward stepwise regression model to predict scores for overall QoL, overall health, and domains (physical health, psychological well-being, social relationships, and environment)—initially for this model, we added all demographic factors to the model (gender, age, place of residence, marital status, being physically active, minutes spent outdoors, meals per day, BMI, RA onset, seropositivity status, smoking, alcohol consumption, scores of PSQI and BDI, and treatment line). Results were reported as the b coefficient and 95% confidence interval (CI). Collinearity diagnostics between included variables were assessed using Variance Inflation Factors (VIF). For categorical variables, VIF values may be inflated due to dummy coding; however, no problematic collinearity was observed among continuous predictors (all VIF < 2). A *p*-value of less than 0.05 was considered statistically significant for all tests.

## 3. Results

### 3.1. Demographic Characteristics of the Participants

A total of 116 patients were included, predominantly middle-aged females with long-standing rheumatoid arthritis. Most participants lived in smaller towns or rural areas and were married. The majority reported reduced physical activity compared to the previous year, with moderate outdoor time and a median BMI of 26 kg/m^2^. Seropositivity was observed in about one-third of patients. Most were non-smokers, consumed little or no alcohol, and were receiving second- or third-line treatment. Only a minority reported good sleep quality, and approximately one in five had moderate to severe depression (Table 1).

### 3.2. WHOQOL-BREF Questionnaire Scoring

The WHOQOL-BREF scores indicated moderate physical health (median = 50), high psychological well-being (66.7), strong social relationships (75), and a supportive environment (68.8). Overall QoL and overall health were both rated with a median score of 75 and 50, respectively (Table 1). The scales’ Cronbach’s standardized alpha demonstrated good reliability (Physical Health α = 0.828, Psychological α = 0.852, Social Relationships α = 0.798, and Environment α = 0.841) [3]. The mean and standard deviation of WHOQOL-BREF domains, along with each item’s correctional and its Cronbach’s Alpha, are shown in Table S1 (Supplementary Material). Regarding the scales of BDI and PSQI, they had Cronbach’s alphas of α = 0.923 and α = 0.739, respectively (Tables S2 and S3—Supplementary Material).

### 3.3. Differences in Demographic Characteristics and WHOQOL-BREF Questionnaire Scoring

The analysis revealed significant differences in quality-of-life scores across demographic characteristics (Table 2). Age was significantly associated with all domains: physical health (*p* = 0.006), psychological well-being (*p* = 0.01), social relationships (*p* = 0.007), environment (*p* = 0.044), overall QoL (*p* = 0.015), and overall health (*p* = 0.02). Marital status also showed significant differences in physical health (*p* < 0.001), psychological well-being (*p* < 0.001), social relationships (*p* = 0.001), and environment (*p* < 0.001). Physical activity levels were significantly associated with overall QoL(*p* < 0.001) and overall health (*p* = 0.017). Smoking status showed a significant difference only in overall QoL(*p* = 0.041). Additionally, having good sleep and mild to borderline depression were significantly associated with better scores in all domains compared to their counterparts. No significant differences were found between the places of residence and alcohol consumption across any domains. Refer to Table S4 (Supplementary Material) for detailed comparisons within each category.

**Figure 1:**
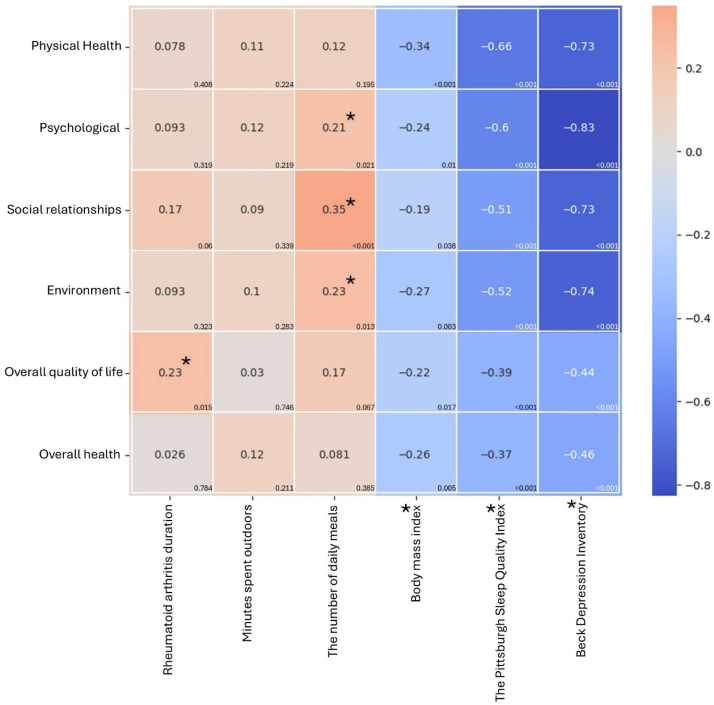
Heatmap of the correlation between RA duration (n = 116), time spent outdoors, number of meals, and BMI, PSQI, and BDI score, and the domains of WHOQOL-BREF (domains: environment, physical health, psychological well-being, social relationships, and overall health, overall QoL). An asterisk denotes significance at *p* < 0.05.

The results also revealed several significant correlations (Figure 1). Physical health was negatively associated with Body Mass Index (BMI) (r = −0.337, *p* < 0.001), indicating that higher BMI corresponds to lower physical health. Psychological well-being showed a positive correlation with the number of daily meals (r = 0.214, *p* = 0.021) and a negative correlation with BMI (r = −0.238, *p* = 0.010). Social Relationships were positively correlated with the number of daily meals (r = 0.349, *p* < 0.001) and negatively correlated with BMI (r = −0.192, *p* = 0.038). Environmental factors had a similar pattern, exhibiting a positive correlation with the number of daily meals (r = 0.230, *p* = 0.013) and a negative correlation with BMI (r = −0.270, *p* = 0.003). Overall QoL was positively correlated with the year rheumatoid arthritis started (r = 0.226, *p* = 0.015) and negatively correlated with BMI (r = −0.221, *p* = 0.017). Finally, overall health negatively correlates with BMI (r = −0.26, *p* = 0.005).

No significant relationships were observed between “Minutes spent outdoors” and any of the six dimensions, and the year rheumatoid arthritis started did not correlate significantly with any measure except Overall QoL. The multivariate backward stepwise regression model enables the prediction of scores for overall QoL, overall health, and specific domains (Table 2; Supplementary Tables S5–S10).

## 4. Discussion

Rheumatoid arthritis (RA) is a chronic inflammatory disease that affects mainly joints, but often co-exists with various extra-articular disorders and may lead to disability, which affects the QoL [21]. Chronic and recurrent pain in the small joints of the hands and feet significantly impacts psychological and sociological areas of the patient’s daily life. The progressive nature of RA leads to the development of disability in about 30% of subjects, which undoubtedly affects the QoL of many patients [2,3,7,22,23,24,25]. Therefore, we studied 116 participants with RA, the majority of whom were women (76.7%) and aged 41 to 60 years (50.9%) (Table 1 and Table S1). The gender distribution corresponds to the epidemiological data, which indicate that RA primarily affects women (a female-to-male ratio is three to one) [26,27]. The age of the analyzed patients is also typical for RA, which develops most often in the third and fourth decades of life, with a peak incidence around the age of 50 [28].

Many studies highlight that the QoL of patients with RA deteriorates with the duration of the disease [2] and is lower than in patients with other joint diseases, such as osteoarthritis [3]. Similarly, in our study, patients under 40 years of age have the highest QoL in physical activity, psychology, social relationships, the general QoL in the environment, and general health (Table S1). This may be because younger people cope better with the disease. After all, they do not have severe joint deformities. Maintaining physical fitness helps preserve mental health and social relationships, ultimately leading to a higher QoL. On the other hand, older age is typically associated with a longer duration of the disease stages, more advanced disease, and permanent, destructive changes that negatively impact various aspects of maintaining well-being. Hence, many studies show that the QoL of RA patients may decrease with the duration of the disease [29,30,31].

Although some data suggest that living in rural areas can be a risk factor for disease development and may affect disease activity [32,33], we did not find such a correlation in our study. However, we did observe the association between marital status and QoL. A close relationship, such as marriage, is considered an important component in the health and functioning of RA patients [34]. Being in a well-adjusted, or at least not distressed, marriage has been shown to have a beneficial effect on health. It provides stable and reliable companionship, a buffer against ongoing stress, and emotional intimacy [35].

Similarly, in our study, analysis of marital status shows that the widower group has the lowest QoL among all the groups analyzed. This is consistent with recently published data showing that marital status (single/divorced/widowed) is independently associated with higher rates of anxiety and depression. Single individuals may face greater inconveniences in their daily lives without the opportunity to discuss problems with their spouses, which increases the risk of anxiety and depression [36]. Similar to other studies on RA [37], the highest QoL was observed in married individuals. This may be because the presence of a supportive person can help individuals cope with the disease, and being aware of the possibility of obtaining support provides a sense of security, which in turn translates into a better QoL [37].

Physical health is another key factor that influences well-being and enhances the QoL. Physically active patients (who had more physical activity than the previous year) had higher overall QoL and overall health. The pain and impaired function affect everyday activities in patients with RA (both early and longstanding) [38,39]. Increased physical activity significantly reduces fatigue and functional disability, improves cardiorespiratory fitness, and increases muscle strength [40,41,42]. Unfortunately, many RA patients, misinterpreting their health status and not knowing the recommendations for physical health, do not practice any sports, and their activity level is much lower than recommended [43]. However, limited physical activity in some of them may also be due to pain, fear of joint damage, and lack of energy [44]. Moreover, many studies confirm that RA patients experience chronic sleep disturbances, fatigue, and pain, which contribute to a more sedentary lifestyle [21,22]. The described relationships are confirmed by the heatmap, which shows that being more physically active than in the previous year improves overall QoL and overall health. In contrast, older age is associated with lower overall health outcomes.

The data describing the impact of alcohol on QoL in RA patients is ambiguous. A recent meta-analysis by Turk et al. confirmed a lower QoL (as measured by the HAQ) in RA patients who consumed alcohol compared with those who did not [45]. However, our study did not find such an association, although patients who consumed alcohol once a week reported the lowest QoL compared with those who drank alcohol less frequently (statistically insignificant association). In the environmental domain, less frequent alcohol consumption (from once a week to never) was associated with higher values of QoL compared with daily drinking (Table 3). Thus, similarly to other studies, moderate alcohol use has a beneficial effect on QoL. Still, there is no consensus whether it is the alcohol or the social context that affects the QoL [46,47]. Moreover, the heatmap indicates that the number of meals consumed is positively correlated with QOL. Interestingly, the more recent onset of RA (e.g., starting in 2010 rather than 1990) was positively associated with social relationships (Figure 1), environment, and overall QoL (Table 3), indicating that individuals with a short disease duration tended to report higher scores in these domains. Similarly, other studies confirm that a shorter RA course is related to better QoL [29,31]. The number of meals consumed was associated with a better social life (Figure 1 and Table 3), which confirms that social relations are an essential element of QoL. The analysis of the psychosocial factors that impact effective management of rheumatoid arthritis (RA) identifies that insufficient support networks are related to ineffective RA management. In contrast, strong social connections emerge as protective influences [48].

Unfortunately, most patients smoked. Smoking has a detrimental effect on many autoimmune diseases, but the association with RA is particularly strong. Smoking distracts from pain and is used as a coping mechanism for musculoskeletal symptoms in RA, making it difficult to quit [49]. This requires decision-making and persistence. A study by Smith et al. showed that RA patients who smoked expressed feelings of insufficiency and fear of not being able to quit, making quitting significantly more difficult [50].

Using a backward stepwise multivariate regression model (heatmap), we showed that the higher the body mass, the worse the parameters in the individual domains (inverse correlation between BMI values and the domains: social relationship, psychological, physical health, environment, overall QoL, and overall health) (Figure 1). We also found that older age (particularly over 40 years) and higher BMI were associated with lower values in the physical health domain (Table 3). Similarly, Nikiphorou et al. reported an inverse association between obesity and QoL in 2386 patients with newly diagnosed RA. They showed that obesity prevalence is rising in early RA and is associated with worse function and health-related QoL [22]. Other studies also confirm that obese individuals are characterized by significantly lower QoL [23] and that high BMI contributes to the pathogenesis of RA [51].

The value of PSQI negatively correlated with all domains of QoL on the heatmap (Figure 1). Thus, the psychological condition is related to sleep quality, and such a relation is particularly strong in RA patients. Moreover, good sleep quality was associated with better physical and psychological health scores and overall QoL (Table 2). Many studies confirm that RA patients experience chronic sleep disruption, fatigue, and pain, which contribute to a more sedentary lifestyle [24,25,52].

In RA patients, joint destruction and pain significantly reduce physical health, contributing not only to decreased mood but also to depression. Epidemiological data show a higher prevalence of anxiety and depressive disorders in RA patients (approximately 25%) compared to the general population (5%) [53]. Furthermore, a high prevalence of major depressive disorders is reported in RA patients (incidence rate ratio 1.46; 95% [CI] 1.35, 1.58) [54]. Our studies also demonstrated an association between RA and depression (Figure 1, Table 3). Depression (as measured by the BDI value) was negatively correlated with the QoL in various domains, including physical health, psychological well-being, social relationships, and overall health (Figure 1). We also found that moderate to severe depression consistently predicted poorer physical health (Table 3). A similar conclusion is provided by the study of Waraich et al., which shows that depression in patients with rheumatoid arthritis is associated with reduced physical function, increased risk of disability, and poor QoL [55]. The associations between RA and depression are reflected in the molecular mechanisms of both diseases. On the one hand, increased IL-6 synthesis is observed in RA, which leads to increased inflammation of the joints and significantly contributes to the development of depression [56,57]. On the other hand, there is a suggestion that other factors besides disease activity and systemic inflammation may cause depression in RA patients [58]. Therefore, the question arises whether depression (as a separate disease entity) primarily affects low physical health values, or whether increased RA activity, secondarily increasing the risk of developing depression, is a key factor determining lower QoL in RA patients.

### Strengths and Limitations

Our study highlighted the complex interplay of psychological, social, and environmental factors, marital status, and social factors that influence QoL in patients with RA. Understanding which factors influencing QoL have the greatest impact on daily functioning can help patients identify supportive elements and adapt lifestyle habits (e.g., modifying social life, treating mood disorders, seeking psychological support). Such changes may help to maintain the balance in everyday life and regain control over their limitations by lifestyle modifications and treatment of co-existing diseases (e.g., sleep disorders or depression). When treating RA, it is crucial to consider current pharmacological treatment recommendations and factors that influence patients’ QoL, as these can impact their well-being and treatment outcomes. Data collection was conducted at a single hospital, so the data may not be representative of the rheumatoid arthritis population in Poland. Therefore, a broad perspective and a comprehensive approach to treatment are necessary, encompassing not only pharmacological guidelines but also methods for improving the QoL of RA patients.

Still, some limitations should be acknowledged. First, its cross-sectional, single-center observational design limits the ability to establish causal relationships between QoL and the assessed factors. Second, the study included only patients with low disease activity or remission, which likely reduced variability in disease-related parameters (e.g., disease activity scores, treatment line, seropositivity status) and may explain why these factors were not significant in the multivariate models. Third, the sample size was moderate and may not fully capture the heterogeneity of the broader RA population. Fourth, the reliance on self-reported questionnaires introduces the possibility of response bias despite the presence of a trained facilitator to ensure comprehension. Fifth, although numerous clinical and demographic variables were initially included in the regression model, the stepwise elimination process may have excluded some parameters that could be relevant in larger or more diverse samples. Finally, the study was conducted in a single geographic and ethnic population (Caucasian patients from one region in Poland), which may limit the generalizability of the findings to other populations and healthcare settings.

## 5. Conclusions

This study evaluates QoL, depression, and quality of sleep in patients with RA, putting an emphasis on the factors beyond conventional treatment—this makes the study unique, and it distinguishes this study from those emphasizing pharmacological treatment. Among the analyzed factors, the strongest positive correlation was observed between the quality of social life and the number of meals eaten. In contrast, the strongest negative correlation was observed between the BDI and physical health, sociological relationships, psychological domains, and sociological domains (as shown in the heat map). Stratifying patients according to their QoL and then identifying environmental factors that can be modified (diet, social relationships, and marital status) can significantly affect the QoL of RA patients. An essential element is the use of modifiable dietary and environmental risk factors, as well as lifestyle modification, in preventing the progression of RA and improving the QoL for patients with this disease.

In the treatment of chronically ill patients, such as those with rheumatoid arthritis, when making therapeutic decisions, it is also worth taking into account the assessment of QoL, paying attention to factors dependent on the disease state (age, marital status, individual patient capabilities, and the degree of obtained social support). Being in a stable marriage can be a supportive factor for people with RA. Individuals living alone may be at greater risk of developing mental health problems, anxiety, and depression, which, if present, should require early psychological or psychiatric intervention through well-designed screening. Given the abundant evidence indicating the beneficial effects of physical activity, RA patients should be encouraged to be physically active.

Since the QoL influences the activity of diseases, focusing on the patient’s psychological, social, and environmental factors may help identify contributors to persistently active diseases. Since strong social support improves health outcomes, patients should be encouraged to maintain meaningful relationships that foster emotional well-being. Therefore, there is a need for a comprehensive biopsychosocial approach that integrates medical, social, and psychological services.

## Figures and Tables

**Table 1:** Characteristics of RA patients.

Group Characteristics of Rheumatoid Arthritis Patients (n = 116)	
Analyzed parameter	IQR; n (%)
Gender	
Female	89 (76.7)
Male	27 (23.3)
Age	
Below 40	25 (21.6)
41–60	59 (50.9)
Over 60	32 (27.6)
Place of residence	
Village	40 (34.5)
City with up to 50 thousand inhabitants	30 (25.9)
City with up to 100 thousand inhabitants	20 (17.2)
A city with over 100 thousand inhabitants	26 (22.4)
Marital status	
Single	21 (18.1)
married	69 (59.5)
In a separation/divorce	10 (8.6)
widower	16 (13.8)
Physically active	
Smaller than the previous year	82 (70.7)
Bigger than the previous year	34 (29.3)
Minutes spent outdoors	120 (90–180)
Meals per day	4 (3–5)
Body mass index	26 (22.7–30.2)
RA onset	2006 (2000–2011)
DAS28-ESR	2.65 (0.26)
Smoking	
Yes	26 (22.4)
No	90 (77.6)
Alcohol consumption	
Everyday	1 (0.9)
Once a week	12 (10.3)
Once a month	9 (7.8)
Occasionally	53 (45.7)
I don’t drink alcohol	41 (35.3)
WHOQOL-BREF	
Physical Health	50 (39.3–60.7)
Psychological	66.7 (54.2–79.2)
Social Relationships	75 (58.3–83.3)
Environment	68.8 (56.3–78.1)
Overall QoL	75 (50–75)
Overall health	50 (25–75)
PSQI	
Good sleep	28 (24.1)
Bad sleep	88 (75.9)
BDI depression	
Extreme depression	1(0.9)
Severe depression	5 (4.3)
Moderate depression	17 (14.7)
Borderline clinical depression	14 (12.1)
Mild mood disturbances	23 (19.8)
Normal	56 (48.3)
Treatment line	
1st	2 (1.7)
2nd	36 (31)
3rd	57 (49.1)
4th	11 (9.5)
5th	9 (7.8)
6th	1 (0.9)

IQR—reported as median, n (%)—frequencies; BDI—the Beck Depression Inventory; PSQI—Pittsburgh Sleep Quality Index; WHOQOL-BREF—World Health Organization Quality of Life Instrument, Short Form.

**Table 2:** Quality of life in various demographic parameters of rheumatoid arthritis patients (n = 116).

	Physical Health	Psychological	Social Relationships	Environment	Overall QoL	Overall Health
Age						
Below 40	57.1 ± 13.7 ^b^	75 (66.7–83.3) ^b^	83.3 (66.7–91.7) ^b^	72 ± 13.4^b^	75 (50–75) ^b^	75 (50–75) ^b^
41–60	47.5 ± 13.4 ^a^	66.7 (54.2–79.2) ^a,b^	75 (58.3–75) ^a^	65.6 ± 14.3 ^a,b^	75 (50–75) ^b^	50 (25–75) ^a,b^
Over 60	45.2 ± 16.6 ^a^	62.5 (50–70.8) ^a^	75 (50–75) ^a^	61.9 ± 17.2 ^a^	50 (50–75) ^a^	50 (25–50) ^a^
*p*-value ^†^	0.015	0.01	0.007	0.044	0.015	0.02
Place of residence						
Village	51.8 (35.7–60.7)	68.8 (52.1–83.3)	75 (54.2–87.5)	68.8 (56.3–78.1)	75 (50–75)	50 (25–75)
A city with up to 50 thousand inhabitants.	51.1 ± 17.3	64.5 ± 15.7	67.2 ± 20.5	68.8 (59.4–78.1)	75 (50–75)	50 (25–75)
A city with up to 100 thousand inhabitants.	46.1 ± 10.9	64.4 ± 13.6	66.7 (58.3–75)	64.2 ± 13.8	62.5 (50–75)	50 (25–62.5)
A city with over 100 thousand inhabitants.	51.4 ± 10.2	68.8 (54.2–75)	69.9 ± 12	68 ± 10.1	75 (50–75)	50 (25–50)
*p*-value	0.474	0.857	0.474	0.824	0.426	0.589
Marital status						
Single	50.8 ± 12.5 ^b^	75 (62.5–79.2) ^b^	70.2 ± 19.5 ^a,b^	69.8 ± 10.1 ^b^	75 (50–75)	50 (25–75)
Married	52.2 ± 13.9 ^b^	66.7 (58.3–79.2) ^b^	75 (66.7–83.3) ^b^	69.6 ± 13.4 ^b^	75 (50–75)	50 (25–75)
In a separation/divorce	45.7 ± 12.8 ^b^	61.7 ± 14.1 ^a,b^	65 ± 20.3 ^a,b^	62.2 ± 13.4 ^a,b^	75 (50–75)	50 (25–50)
Widower	34.4 ± 15.6 ^a^	49.2 ± 14.5 ^a^	45.9 (33.3–70.9) ^a^	50 (29.7–61) ^a^	50 (50–75)	50 (25–50)
*p*-value ^†^	<0.001	<0.001	0.001	<0.001	0.059	0.066
Physically active						
Smaller than the previous year	47.7 ± 14.8	62.5 (54.2–79.2)	70.9 (58.3–83.3)	64.1 (56.3–78.1)	62.5 (50–75)	50 (25–50)
Bigger than the previous year	52 ± 15	70.8 (58.3–79.2)	75 (66.7–75)	69.8 ± 11.7	75 (75–75)	50 (50–75)
*p*-value	0.16	0.157	0.384	0.118	<0.001	0.017
Smoking						
Yes	47.5 ± 17.6	62.5 (50–75)	63.8 ± 19.3	60.7 ± 18.9	50 (50–75)	50 (25–50)
No	49.4 ± 14.2	66.7 (54.2–79.2)	75 (58.3–83.3)	68.8 (59.4–78.1)	75 (50–75)	50 (25–75)
*p*-value	0.583	0.145	0.073	0.065	0.041	0.582
Alcohol consumption						
Everyday						
Once a week	41.7 ± 24.2	55.6 ± 21	61.8 ± 22.6	54.7 ± 21.4	50 ± 23.8	50 (50–62.5)
Once a month	55.9 ± 13.8	79.2 (70.8–79.2)	79.6 ± 21.7	71.2 ± 14.2	75 (75–75)	50 (50–75)
Occasionally	48.7 ± 13.9	66.7 (54.2–79.2)	75 (58.3–75)	67.6 ± 13.8	75 (50–75)	50 (25–75)
Don’t drink	50 (46.4–60.7)	66.7 (54.2–79.2)	75 (66.7–83.3)	67 ± 12.9	75 (50–75)	50 (25–75)
*p*-value	0.31	0.171	0.094	0.209	0.051	0.518
PSQI						
Good sleep	59.9 ± 12	79.2 (69.775–80.225)	75 (66.7–83.3)	74.8 ± 12.3	75 (68.75–75)	50 (50–75)
Bad sleep	46.4 (35.7–57.1)	62.5 (50–75)	75 (58.3–75)	64.05 (56.3–75)	75 (50–75)	50 (25–75)
*p*-value	<0.001	<0.001	0.036	0.001	0.015	0.005
BDI						
Moderate to severe	30.9 ± 13.7	45.3 ± 11.5	46.4 ± 14.6	47 ± 14.6	50 (50–50)	25 (25–50)
Mild to borderline	53.4 ± 11.5	70.8 (62.5–79.2)	75 (66.7–83.3)	70.6 ±11.5	75 (50–75)	50 (25–75)
*p*-value	<0.001	<0.001	<0.001	<0.001	<0.001	0.002
	Physical Health	Psychological	Social Relationships	Environment	Overall QoL	Overall health
Treatment line						
1st line	58.9 (57.1–60.7)	79.2 (79.2–79.2)	87.5 (75–100)	76.6 (75–78.1)	62.5 (50–75)	50 (25–75)
2nd line	46.6 + 17.3	62.7 + 17.4	75 (54.2–79.2)	66.2 + 18.5	75 (50–75)	50 (25–62.5)
3rd line	49.7 + 13.3	66.7 (54.2–79.2)	75 (58.3–83.3)	64.3 + 13.8	75 (50–75)	50 (25–75)
4th line	50 + 19.2	75 (58.3–83.3)	73.5 + 23.3	78.1 (56.3–78.1)	75 (50–75)	75 (50–75)
5th line	49.7 + 12.2	68.6 + 8.7	69.5 + 11.8	69.5 + 12	75 (50–75)	47.3 + 19.6
*p*-value	0.512	0.467	0.578	0.396	0.933	0.466

^†^* p*-value of significant differences between the groups. Post hoc analysis results are denoted with letters ^a^ and ^b^ in each category. Numbers with the same letter indicate comparable results, while the letter ^b^ denotes statistical significance compared to the group labeled with ^a^. All results are reported at *p*-value < 0.05.

**Table 3:** Prediction of QoL parameters by a multivariate backward stepwise regression model in various WHO-BREF domains ^†^.

	Prediction Parameters of QoL (n = 116)							
Analyzed Parameter	b Coeff.	b Error	−95% CI	+95% CI	t-Stat.	*p*-Value	b Stand.	b Stand. Error
Physical Health								
Age	−2.959	1.479	−5.89	−0.027	−2.000	0.048	-0.139	0.069
BMI	−0.617	0.201	−1.016	−0.218	−3.063	0.003	−0.212	0.069
good sleep	8.531	2.44	3.695	13.367	3.496	0.001	0.245	0.07
moderate to severe depression	−18.394	2.614	−23.573	−13.215	−7.038	<0.001	−0.493	0.07
Psychological								
BMI	−0.62	0.212	−1.04	−0.201	−2.93	0.004	−0.205	0.07
good sleep	5.859	2.607	0.694	11.024	2.248	0.027	0.162	0.072
moderate to severe depression	−21.477	2.823	−27.072	−15.883	−7.607	<0.001	−0.554	0.073
Social Relationships								
RA durations	0.437	0.154	0.131	0.742	2.831	0.006	0.193	0.068
Meals per day	4.213	1.338	1.562	6.865	3.149	0.002	0.22	0.07
BMI	−0.477	0.252	−0.976	0.023	−1.892	0.061	−0.13	0.069
moderate to severe depression	−25.5	3.303	−32.045	−18.955	−7.72	<0.001	−0.543	0.07
Environment								
RA durations	0.295	0.126	0.044	0.545	2.333	0.022	0.16	0.069
Alcohol consumption, reference [everyday]								
Once a week	18.704	11.7	−4.489	41.897	1.599	0.113	0.374	0.234
Once a month	23.047	12.032	−0.805	46.898	1.915	0.058	0.405	0.212
Occasionally	20.948	11.543	−1.934	43.83	1.815	0.072	0.686	0.378
I don’t drink alcohol	20.971	11.568	−1.961	43.902	1.813	0.073	0.659	0.363
BMI	−0.65	0.209	−1.064	−0.236	−3.113	0.002	-0.218	0.07
good sleep	5.656	2.519	0.662	10.65	2.245	0.027	0.159	0.071
Overall QoL								
RA durations	0.544	0.176	0.196	0.892	3.099	0.002	0.262	0.085
Being more physically active than the previous year	13.288	3.184	6.981	19.596	4.174	<0.001	0.353	0.085
Overall health								
Minutes spent outdoors	0.042	0.023	−0.004	0.088	1.805	0.074	0.158	0.088
BMI	−0.859	0.354	−1.561	−0.156	−2.423	0.017	−0.214	0.088
Good sleep	10.141	4.295	1.63	18.652	2.361	0.02	0.212	0.09
moderate to severe depression	−10.048	4.657	−19.276	−0.821	−2.158	0.033	−0.195	0.091

BMI—body mass index. ^†^ Results for collinearity are in Table S4.

## Data Availability

No datasets were generated during the current study. The data supporting this study’s findings are available from the corresponding author upon reasonable request.

## Supplementary Materials

The following supporting information can be downloaded at https://www.mdpi.com/article/10.3390/jcm14248925/s1, Table S1. Analysis of WHO-BREF domains in the group of rheumatoid arthritis patients; Table S2. Sleep characteristics estimated by the Pittsburgh Sleep Quality Index; Table S3. Beck Depression Inventory characteristics; Table S4. Collinearity diagnostics; Table S5. Prediction of QoL parameters by a multivariate regression model in the Physical Health WHO-BREF domain; Table S6. Prediction of QoL parameters by a multivariate regression model in the Psychological WHO-BREF domain; Table S7. Prediction of QoL parameters by a multivariate regression model in the Social Relationships WHO-BREF domain; Table S8. Prediction of QoL parameters by a multivariate regression model in the Environment WHO-BREF domain; Table S9. Prediction of QoL parameters by a multivariate regression model in the Overall QoL WHO-BREF domain; Table S10. Prediction of QoL parameters by a multivariate regression model in the Overall Health WHO-BREF domain.

## Abbreviations

The following abbreviations are used in this manuscript:

BDI	Beck Depression Inventory
BMI	Body Mass Index
IQR	reported as median
PSQI	Pittsburgh Sleep Quality Index
QoL	Quality of Life
RA	Rheumatoid arthritis
WHOQOL-BREF	World Health Organization Quality of Life Instrument, Short Form
