# Supplementary material for: Quality of Life in Long-Standing Rheumatoid Arthritis: What Can Help Apart from Treatment? A Single-Center Cross-Sectional Observational Study
Source: J Clin Med. 2025 Dec 17;14(24):8925. doi: 10.3390/jcm14248925 (PMC12733405; doi:10.3390/jcm14248925)
Supplement: Supplementary file 1 [file jcm-14-08925-s001.zip › jcm-4039347-supplementary.pdf]

Supplementary materials for

Quality of Life in Long-Standing Rheumatoid Arthritis – What Can Help Apart from Treatment? A Single-Center Cross-Sectional Observational Study

Bogna Grygiel-Górniak[1,*], 1Ewelina Kowynia[1], Mohamed Abouzid[2], Anna Kwaśniewska[3], Maria Joks[4], Natalia Majewska[5], Włodzimierz Samborski[1]

[1] Poznan University of Medical Sciences, Department of Rheumatology, Rehabilitation and Internal Diseases, Poznań, Poland bgrygiel@ump.edu.pl
[2] Poznan University of Medical Sciences, Department of Physical Pharmacy and Pharmacokinetics, Faculty of Pharmacy, Poznań, Poland
[3] Rheumatological Centre in Śrem, Poland
[4] Poznan University of Medical Sciences, Department of Rheumatology, Rehabilitation and Internal Diseases, Rheumatology Research Group, Poznań, Poland
[5] Poznan University of Medical Sciences, Department of Radiology, Poznań, Poland
* Correspondence: bgrygiel@ump.edu.pl; ORCID: 0000-0002-3438-0764

Table S1. Analysis of WHO-BREF domains in the group of rheumatoid arthritis patients

Domain	Analyzed parameters		
	**Mean (standard deviation) N=116**	**Item-total scale correlation**	**Cronbach's Alpha, if the item is deleted**
Overall QoL	64.2 (17.13)[†]		
Overall health	50 (20.5)[†]		
**Physical Health**	20.71 (4.18)[&]<br>49 (14.87)[‡]	0.404[β]	0.828[α]
Pain	2.38 (0.81)	0.359	0.837
Dependence on medical aids	2.15 (0.82)	0.273	0.849
Energy	3.33 (0.76)	0.662	0.792
Mobility	3.51 (0.86)	0.646	0.793
Sleep and rest	3.2 (0.93)	0.591	0.802
Activities of daily living	3.11 (0.85)	0.766	0.772
Work capacity	3.03 (0.93)	0.75	0.772
**Psychological**	21.56 (3.72)[&]<br>64.8 (15.45)[‡]	0.5β	0.852α
Positive feelings	3.36 (0.69)	0.67	0.824
Personal belief	3.89 (0.79)	0.742	0.808
Concentration	3.75 (0.73)	0.525	0.846
Bodily image	3.63 (1.03)	0.594	0.844
Self-esteeem	3.49 (0.86)	0.766	0.801
Negative feelings	3.44 (0.76)	0.58	0.837
**Social Relationships**	11.36 (2.26)[&]<br>69.7 (18.7)[‡]	0.589[β]	0.798[α]
Personal relationship	3.97 (0.75)	0.668	0.72
Sexual activity	3.4 (1.05)	0.631	0.767
Social support	4 (0.86)	0.672	0.696
**Environment**	29.1 (4.89)[&]<br>65.9 (15.22)[‡]	0.411[β]	0.841[α]
Security	3.78 (0.7)	0.637	0.819
Physical environment	3.67 (0.8)	0.604	0.82
Financial support	3.45 (1.14)	0.614	0.82
Accessibility of information	3.78 (0.84)	0.581	0.822
Leisure activity	3.47 (0.99)	0.704	0.804
Home environment	4.22 (0.78)	0.699	0.809
Home care	3.27 (0.95)	0.399	0.846
Transport	3.46 (0.83)	0.439	0.838

[†] reported on a 0- 100 scale
[‡] reported on the raw scale and the 0- 100 scale
[α] the domain Cronbach's alpha
[β] average correlation between pairs of items

Abbreviations: QoL – quality of life

Table S2. Sleep characteristics estimated by the Pittsburgh Sleep Quality Index (PSQI)

Domain	Mean (standard deviation) n=116	Item-total scale correlation	Cronbach's Alpha, if the item is deleted
Subjective sleep quality	5.89 (3)	0.548	0.689
Sleep latency	6.48 (2.97)	0.453	0.713
Sleep duration	7.53 (3.22)	0.277	0.741
Sleep efficiency	7.72 (3.1)	0.374	0.725
Sleep disturbance	6.82 (3.08)	0.588	0.691
Use of sleep medication	7.35 (2.74)	0.498	0.709
Daytime dysfunction	6.92 (2.97)	0.563	0.684
PSQI Scale	average correlation	0.304	$\alpha = 0.739$

Table S3. Beck Depression Inventory (BDI) characteristics

Question No.	Mean (standard deviation)	Item-total scale correlation	Cronbach's Alpha, if the item is deleted
1	12.34 (9.13)	0.777	0.916
2	12.02 (9.28)	0.518	0.921
3	12.46 (9.18)	0.637	0.919
4	12.29 (9.04)	0.715	0.917
5	12.39 (9.18)	0.688	0.918
6	12.60 (9.26)	0.465	0.922
7	12.41 (9.20)	0.727	0.918
8	12.42 (9.18)	0.749	0.917
9	12.79 (9.56)	0.311	0.924
10	12.49 (9.19)	0.628	0.919
11	12.21 (9.05)	0.679	0.918
12	12.20 (9.11)	0.725	0.917
13	12.21 (9.12)	0.743	0.917
14	11.95 (9.02)	0.659	0.918
15	12.13 (9.12)	0.774	0.916
16	11.97 (9.06)	0.720	0.917
17	11.97 (9.31)	0.549	0.921
18	12.39 (9.43)	0.290	0.925
19	12.62 (9.68)	–0.077	0.930
20	11.85 (9.17)	0.479	0.923
21	11.71 (9.05)	0.528	0.923
BDI Scale	average correlation	0.367	$\alpha = 0.923$

Table S4. Collinearity diagnostics using Variance Inflation Factors for a multivariate backward stepwise regression model

Analyzed variable	partial	semipartial	tolerance	R^2	VIF	t stat.	p-value
			**Physical Health**				
**Age**	-0.187	-0.133	0.920	0.080	1.087	-2.000	0.048
**BMI**	-0.279	-0.204	0.927	0.073	1.078	-3.063	0.003
**good sleep**	0.315	-0.233	0.901	0.099	1.110	3.496	0.001
**moderate to severe depression**	-0.555	-0.469	0.905	0.095	1.106	-7.038	<0.001
			**Psychological**				
**BMI**	-0.267	-0.203	0.979	0.021	1.021	-2.930	0.004
**good sleep**	0.208	-0.156	0.921	0.079	1.086	2.248	0.027
**moderate to severe depression**	-0.584	-0.527	0.905	0.095	1.106	-7.607	<0.001
			**Social Relationships**				
**RA durations**	0.259	-0.192	0.997	0.003	1.003	2.831	0.006
**Meals per day**	0.286	-0.214	0.946	0.054	1.057	3.149	0.002
**BMI**	-0.177	-0.129	0.975	0.025	1.026	-1.892	0.061
**moderate to severe depression**	-0.591	-0.525	0.933	0.067	1.072	-7.720	<0.001
			**Environment**				
**RA durations**	0.188	-0.159	0.969	0.031	1.032	1.991	0.049
**Alcohol consumption, reference [everyday]**							
**Once a week**	0.185	-0.157	0.084	0.916	11.886	1.960	0.053
**Once a month**	0.250	-0.215	0.105	0.895	9.487	2.686	0.008
**Occasionally**	0.240	-0.206	0.033	0.967	30.321	2.574	0.011
**I don't drink alcohol**	0.246	-0.212	0.036	0.964	27.924	2.643	0.009
**BMI**	-0.277	-0.240	0.930	0.070	1.075	-2.994	0.003
**good sleep**	0.324	-0.285	0.981	0.019	1.020	3.559	0.001
			**Overall QoL**				
**RA durations**	0.280	-0.262	1.000	0.000	1.000	3.099	0.002
**Being more physically active than the previous year**	0.365	-0.353	1.000	0.000	1.000	4.174	<0.001
			**Overall health**				
**Minutes spent outdoors**	0.169	-0.155	0.963	0.037	1.039	1.805	0.074
**BMI**	-0.224	-0.208	0.946	0.054	1.057	-2.423	0.017
**Good sleep**	0.219	-0.203	0.919	0.081	1.089	2.361	0.020
**moderate to severe depression**	-0.201	-0.185	0.900	0.100	1.111	-2.158	0.033

Abbreviations: BMI – body mass index; RA – rheumatoid arthritis; QoL – quality of life

Table S5. Prediction of QoL parameters by a multivariate regression model in the Physical Health WHO-BREF domain.

	b coeff.	b error	-95% CI	+95% CI	t stat.	p-value	b stand.	b stand. error
**Age**	-1.475	1.961	-5.369	2.419	-0.752	0.454	-0.069	0.092
**Place of residence (ref: village)**								
**City with up to 50,000 inhabitants**	3.577	2.710	-1.803	8.958	1.320	0.190	0.105	0.080
**City with up to 100,000 inhabitants**	-3.036	3.160	-9.311	3.239	-0.961	0.339	-0.077	0.080
**City with over 100,000 inhabitants**	0.982	3.014	-5.003	6.968	0.326	0.745	0.028	0.085
**Marital status (ref: single)**								
**Married**	4.400	2.881	-1.322	10.121	1.527	0.130	0.145	0.095
**Separated/Divorced**	3.268	4.466	-5.602	12.137	0.732	0.466	0.062	0.084
**Widowed**	-3.096	4.634	-12.298	6.107	-0.668	0.506	-0.072	0.107
**RA onset**	0.129	0.140	-0.150	0.407	0.916	0.362	0.071	0.078
**Physically active**	-2.415	2.440	-7.261	2.431	-0.990	0.325	-0.074	0.075
**Minutes spent outdoors**	0.017	0.014	-0.011	0.044	1.200	0.233	0.087	0.072
**Meals per day**	1.247	1.136	-1.008	3.502	1.098	0.275	0.082	0.075
**Smoking**	-4.415	2.890	-10.155	1.325	-1.527	0.130	-0.124	0.081
**Alcohol consumption (ref: none)**								
**Everyday alcohol use**	-10.668	11.554	-33.612	12.276	-0.923	0.358	-0.218	0.237
**Weekly alcohol use**	-8.063	12.015	-31.922	15.796	-0.671	0.504	-0.145	0.216
**Monthly alcohol use**	-12.030	11.721	-35.306	11.246	-1.026	0.307	-0.403	0.393
**Occasional alcohol use**	-8.816	11.863	-32.374	14.742	-0.743	0.459	-0.283	0.381
**BMI**	-0.770	0.225	-1.216	-0.324	-3.427	0.001	-0.265	0.077
**PSQI**	9.684	2.626	4.470	14.898	3.688	0.000	0.279	0.076
**BDI**	-16.492	2.932	-22.315	-10.669	-5.624	<0.000001	-0.442	0.079
**bDMARD**	0.556	3.041	-5.482	6.595	0.183	0.855	0.019	0.102
**Treatment line**	1.025	1.599	-2.150	4.201	0.641	0.523	0.064	0.100
**aCCP and RF**	-3.229	2.376	-7.947	1.489	-1.359	0.177	-0.099	0.073

Abbreviations: BMI – body mass index; RA – rheumatoid arthritis; QoL – quality of life; PSQI – Pittsburgh Sleep Quality Index; BDI – Beck Depression Inventory; bDMARD – biologic disease-modifying antirheumatic drugs; aCCP – anti-cyclic citrullinated peptide; RF – rheumatoid factor

Table S6. Prediction of QoL parameters by a multivariate regression model in the Psychological WHO-BREF domain.

	b coeff.	b error	-95% CI	+95% CI	t stat.	p-value	b stand.	b stand. error
**Age**	1.159	2.114	-3.040	5.357	0.548	0.585	0.052	0.096
**Place of residence (ref: village)**								
**City with up to 50,000 inhabitants**	-2.452	2.921	-8.254	3.349	-0.839	0.403	-0.070	0.083
**City with up to 100,000 inhabitants**	-2.978	3.407	-9.743	3.788	-0.874	0.384	-0.073	0.083
**City with over 100,000 inhabitants**	-7.228	3.250	-13.682	-0.774	-2.224	0.029	-0.195	0.088
**Marital status (ref: single)**								
**Married**	-3.027	3.106	-9.195	3.142	-0.974	0.332	-0.096	0.099
**Separated/Divorced**	-1.954	4.816	-11.517	7.609	-0.406	0.686	-0.036	0.087
**Widowed**	-11.892	4.997	-21.814	-1.970	-2.380	0.019	-0.265	0.112
**RA onset**	0.108	0.151	-0.192	0.409	0.716	0.476	0.058	0.081
**Physically active**	-0.135	2.631	-5.360	5.090	-0.051	0.959	-0.004	0.078
**Minutes spent outdoors**	0.017	0.015	-0.012	0.047	1.167	0.246	0.088	0.075
**Meals per day**	2.028	1.224	-0.403	4.460	1.657	0.101	0.128	0.077
**Smoking**	1.156	3.116	-5.033	7.344	0.371	0.712	0.031	0.084
**Alcohol consumption (ref: none)**								
**Everyday alcohol use**	1.314	12.457	-23.424	26.052	0.105	0.916	0.026	0.246
**Weekly alcohol use**	6.263	12.954	-19.461	31.988	0.483	0.630	0.108	0.224
**Monthly alcohol use**	-0.642	12.638	-25.738	24.453	-0.051	0.960	-0.021	0.408
**Occasional alcohol use**	-0.316	12.791	-25.716	25.084	-0.025	0.980	-0.010	0.396
**BMI**	-0.535	0.242	-1.016	-0.055	-2.211	0.029	-0.177	0.080
**PSQI**	5.528	2.831	-0.093	11.150	1.953	0.054	0.153	0.078
**BDI**	-19.871	3.162	-26.150	-13.593	-6.285	<0.000001	-0.513	0.082
**bDMARD**	-0.106	3.279	-6.617	6.404	-0.032	0.974	-0.003	0.106
**Treatment line**	0.693	1.724	-2.731	4.116	0.402	0.689	0.042	0.103
**aCCP and RF**	-1.027	2.562	-6.114	4.060	-0.401	0.689	-0.030	0.075

Abbreviations: BMI – body mass index; RA – rheumatoid arthritis; QoL – quality of life; PSQI – Pittsburgh Sleep Quality Index; BDI – Beck Depression Inventory; bDMARD – biologic disease-modifying antirheumatic drugs; aCCP – anti-cyclic citrullinated peptide; RF – rheumatoid factor

Table S7. Prediction of QoL parameters by a multivariate regression model in the Social Relationships WHO-BREF domain.

	b coeff.	b error	-95% CI	+95% CI	t stat.	p-value	b stand.	b stand. error
**Age**	-2.861	2.417	-7.660	1.939	-1.184	0.240	-0.107	0.090
**Place of residence (ref: village)**								
**City with up to 50,000 inhabitants**	-5.403	3.340	-12.035	1.229	-1.618	0.109	-0.126	0.078
**City with up to 100,000 inhabitants**	-5.350	3.895	-13.084	2.383	-1.374	0.173	-0.108	0.079
**City with over 100,000 inhabitants**	-7.476	3.715	-14.854	-0.098	-2.012	0.047	-0.167	0.083
**Marital status (ref: single)**								
**Married**	5.836	3.551	-1.215	12.888	1.644	0.104	0.153	0.093
**Separated/Divorced**	6.537	5.505	-4.395	17.469	1.188	0.238	0.098	0.083
**Widowed**	-2.649	5.712	-13.991	8.693	-0.464	0.644	-0.049	0.105
**RA onset**	0.268	0.173	-0.076	0.612	1.548	0.125	0.118	0.076
**Physically active**	-0.160	3.008	-6.133	5.813	-0.053	0.958	-0.004	0.073
**Minutes spent outdoors**	0.014	0.017	-0.020	0.048	0.814	0.418	0.058	0.071
**Meals per day**	4.012	1.400	1.233	6.792	2.867	0.005	0.209	0.073
**Smoking**	3.206	3.563	-3.869	10.280	0.900	0.371	0.071	0.079
**Alcohol consumption (ref: none)**								
**Everyday alcohol use**	1.505	14.240	-26.773	29.784	0.106	0.916	0.024	0.232
**Weekly alcohol use**	5.940	14.808	-23.466	35.346	0.401	0.689	0.085	0.212
**Monthly alcohol use**	-7.024	14.446	-35.712	21.663	-0.486	0.628	-0.187	0.385
**Occasional alcohol use**	-2.223	14.621	-31.258	26.812	-0.152	0.879	-0.057	0.374
**BMI**	-0.633	0.277	-1.183	-0.084	-2.288	0.024	-0.173	0.076
**PSQI**	2.470	3.236	-3.956	8.896	0.763	0.447	0.056	0.074
**BDI**	-25.359	3.614	-32.536	-18.182	-7.017	<0.000001	-0.540	0.077
**bDMARD**	-5.872	3.748	-13.314	1.571	-1.567	0.121	-0.157	0.100
**Treatment line**	0.428	1.971	-3.486	4.342	0.217	0.829	0.021	0.098
**aCCP and RF**	2.358	2.928	-3.457	8.173	0.805	0.423	0.057	0.071

Abbreviations: BMI – body mass index; RA – rheumatoid arthritis; QoL – quality of life; PSQI – Pittsburgh Sleep Quality Index; BDI – Beck Depression Inventory; bDMARD – biologic disease-modifying antirheumatic drugs; aCCP – anti-cyclic citrullinated peptide; RF – rheumatoid factor

Table S8. Prediction of QoL parameters by a multivariate regression model in the Environment WHO-BREF domain.

	b coeff.	b error	-95% CI	+95% CI	t stat.	p-value	b stand.	b stand. error
**Age**	0.997	1.994	-2.962	4.956	0.500	0.618	0.046	0.092
**Place of residence (ref: village)**								
**City with up to 50,000 inhabitants**	0.812	2.754	-4.658	6.282	0.295	0.769	0.023	0.079
**City with up to 100,000 inhabitants**	-2.364	3.212	-8.743	4.014	-0.736	0.464	-0.059	0.080
**City with over 100,000 inhabitants**	-2.110	3.064	-8.195	3.975	-0.689	0.493	-0.058	0.084
**Marital status (ref: single)**								
**Married**	0.906	2.929	-4.910	6.722	0.309	0.758	0.029	0.094
**Separated/Divorced**	0.004	4.540	-9.013	9.020	0.001	0.999	0.000	0.084
**Widowed**	-11.980	4.711	-21.335	-2.625	-2.543	0.013	-0.272	0.107
**RA onset**	0.231	0.143	-0.052	0.515	1.621	0.108	0.126	0.077
**Physically active**	-0.429	2.481	-5.356	4.497	-0.173	0.863	-0.013	0.074
**Minutes spent outdoors**	0.011	0.014	-0.017	0.039	0.798	0.427	0.057	0.072
**Meals per day**	1.885	1.154	-0.407	4.177	1.633	0.106	0.121	0.074
**Smoking**	0.269	2.938	-5.565	6.104	0.092	0.927	0.007	0.081
**Alcohol consumption (ref: none)**								
**Everyday alcohol use**	7.495	11.745	-15.828	30.819	0.638	0.525	0.150	0.235
**Weekly alcohol use**	13.406	12.214	-10.848	37.660	1.098	0.275	0.236	0.215
**Monthly alcohol use**	8.823	11.915	-14.838	32.485	0.741	0.461	0.289	0.390
**Occasional alcohol use**	8.675	12.059	-15.273	32.622	0.719	0.474	0.273	0.379
**BMI**	-0.579	0.228	-1.033	-0.126	-2.538	0.013	-0.195	0.077
**PSQI**	6.383	2.669	1.083	11.683	2.391	0.019	0.180	0.075
**BDI**	-16.381	2.981	-22.300	-10.461	-5.495	<0.000001	-0.429	0.078
**bDMARD**	-3.412	3.091	-9.550	2.726	-1.104	0.273	-0.112	0.102
**Treatment line**	1.952	1.626	-1.276	5.180	1.201	0.233	0.119	0.099
**aCCP and RF**	-2.897	2.415	-7.693	1.899	-1.200	0.233	-0.087	0.072

Abbreviations: BMI – body mass index; RA – rheumatoid arthritis; QoL – quality of life; PSQI – Pittsburgh Sleep Quality Index; BDI – Beck Depression Inventory; bDMARD – biologic disease-modifying antirheumatic drugs; aCCP – anti-cyclic citrullinated peptide; RF – rheumatoid factor

Table S9. Prediction of QoL parameters by a multivariate regression model in the Overall QoL WHO-BREF domain.

	b coeff.	b error	-95% CI	+95% CI	t stat.	p-value	b stand.	b stand. error
**Age**	0.651	2.625	-4.562	5.864	0.248	0.805	0.027	0.107
**Place of residence (ref: village)**								
**City with up to 50,000 inhabitants**	0.913	3.627	-6.289	8.116	0.252	0.802	0.023	0.093
**City with up to 100,000 inhabitants**	-2.939	4.230	-11.339	5.460	-0.695	0.489	-0.065	0.093
**City with over 100,000 inhabitants**	-8.618	4.035	-16.630	-0.605	-2.136	0.035	-0.210	0.098
**Marital status (ref: single)**								
**Married**	-3.182	3.857	-10.840	4.476	-0.825	0.411	-0.091	0.111
**Separated/Divorced**	6.179	5.979	-5.694	18.051	1.033	0.304	0.101	0.098
**Widowed**	-3.923	6.203	-16.241	8.395	-0.632	0.529	-0.079	0.125
**RA onset**	0.433	0.188	0.060	0.806	2.303	0.024	0.209	0.091
**Physically active**	10.247	3.267	3.760	16.734	3.137	0.002	0.272	0.087
**Minutes spent outdoors**	0.003	0.019	-0.034	0.040	0.172	0.864	0.014	0.084
**Meals per day**	1.895	1.520	-1.124	4.913	1.246	0.216	0.108	0.087
**Smoking**	2.190	3.869	-5.494	9.873	0.566	0.573	0.053	0.094
**Alcohol consumption (ref: none)**								
**Everyday alcohol use**	-12.726	15.466	-43.438	17.986	-0.823	0.413	-0.226	0.275
**Weekly alcohol use**	-1.456	16.083	-33.392	30.481	-0.091	0.928	-0.023	0.251
**Monthly alcohol use**	-8.562	15.690	-39.719	22.594	-0.546	0.587	-0.249	0.456
**Occasional alcohol use**	-6.201	15.880	-37.735	25.332	-0.391	0.697	-0.173	0.443
**BMI**	-0.080	0.301	-0.677	0.517	-0.267	0.790	-0.024	0.090
**PSQI**	2.119	3.515	-4.860	9.099	0.603	0.548	0.053	0.088
**BDI**	-14.096	3.925	-21.891	-6.302	-3.591	0.001	-0.328	0.091
**bDMARD**	5.020	4.070	-3.063	13.103	1.233	0.221	0.147	0.119
**Treatment line**	0.456	2.140	-3.794	4.707	0.213	0.832	0.025	0.116
**aCCP and RF**	-5.663	3.180	-11.978	0.652	-1.781	0.078	-0.151	0.085

Abbreviations: BMI – body mass index; RA – rheumatoid arthritis; QoL – quality of life; PSQI – Pittsburgh Sleep Quality Index; BDI – Beck Depression Inventory; bDMARD – biologic disease-modifying antirheumatic drugs; aCCP – anti-cyclic citrullinated peptide; RF – rheumatoid factor

Table S10. Prediction of QoL parameters by a multivariate regression model in the Overall Health WHO-BREF domain.

	b coeff.	b error	-95% CI	+95% CI	t stat.	p-value	b stand.	b stand. error
**Age**	-1.383	3.466	-8.267	5.500	-0.399	0.691	-0.047	0.118
**Place of residence (ref: village)**								
**City with up to 50,000 inhabitants**	1.305	4.789	-8.205	10.816	0.273	0.786	0.028	0.102
**City with up to 100,000 inhabitants**	0.112	5.585	-10.979	11.203	0.020	0.984	0.002	0.103
**City with over 100,000 inhabitants**	-8.756	5.328	-19.336	1.824	-1.643	0.104	-0.178	0.108
**Marital status (ref: single)**								
**Married**	7.123	5.092	-2.990	17.235	1.399	0.165	0.171	0.122
**Separated/Divorced**	0.030	7.894	-15.647	15.707	0.004	0.997	0.000	0.108
**Widowed**	2.256	8.191	-14.010	18.522	0.275	0.784	0.038	0.138
**RA onset**	-0.070	0.248	-0.562	0.423	-0.280	0.780	-0.028	0.100
**Physically active**	6.850	4.314	-1.716	15.416	1.588	0.116	0.152	0.096
**Minutes spent outdoors**	0.040	0.025	-0.008	0.089	1.643	0.104	0.153	0.093
**Meals per day**	1.936	2.007	-2.050	5.921	0.964	0.337	0.092	0.096
**Smoking**	3.112	5.109	-7.034	13.257	0.609	0.544	0.063	0.104
**Alcohol consumption (ref: none)**								
**Everyday alcohol use**	-18.145	20.422	-58.699	22.409	-0.888	0.377	-0.270	0.303
**Weekly alcohol use**	-24.591	21.236	-66.762	17.580	-1.158	0.250	-0.321	0.277
**Monthly alcohol use**	-34.263	20.717	-75.404	6.878	-1.654	0.102	-0.833	0.503
**Occasional alcohol use**	-30.247	20.968	-71.886	11.392	-1.443	0.153	-0.705	0.489
**BMI**	-0.908	0.397	-1.697	-0.120	-2.288	0.024	-0.226	0.099
**PSQI**	8.660	4.641	-0.555	17.876	1.866	0.065	0.181	0.097
**BDI**	-11.222	5.183	-21.515	-0.930	-2.165	0.033	-0.218	0.101
**bDMARD**	5.250	5.375	-5.424	15.923	0.977	0.331	0.128	0.131
**Treatment line**	-0.776	2.826	-6.389	4.837	-0.275	0.784	-0.035	0.128
**aCCP and RF**	-3.386	4.199	-11.725	4.953	-0.806	0.422	-0.075	0.093

Abbreviations: BMI – body mass index; RA – rheumatoid arthritis; QoL – quality of life; PSQI – Pittsburgh Sleep Quality Index; BDI – Beck Depression Inventory; bDMARD – biologic disease-modifying antirheumatic drugs; aCCP – anti-cyclic citrullinated peptide; RF – rheumatoid factor
